# Increased Mitochondrial DNA Copy Number in Occupations Associated with Low-Dose Benzene Exposure

**DOI:** 10.1289/ehp.1103979

**Published:** 2011-10-17

**Authors:** Michele Carugno, Angela Cecilia Pesatori, Laura Dioni, Mirjam Hoxha, Valentina Bollati, Benedetta Albetti, Hyang-Min Byun, Matteo Bonzini, Silvia Fustinoni, Pierluigi Cocco, Giannina Satta, Mariagrazia Zucca, Domenico Franco Merlo, Massimo Cipolla, Pier Alberto Bertazzi, Andrea Baccarelli

**Affiliations:** 1 Department of Occupational and Environmental Health, Università degli Studi di Milano, Milan, Italy; 2 Fondazione IRCCS Ca’ Granda – Ospedale Maggiore Policlinico, Milan, Italy; 3 Department of Environmental Health, Harvard School of Public Health, Boston, Massachusetts, USA; 4 Department of Clinical and Biological Sciences, University of Insubria, Varese, Italy; 5 Department of Biomedical Sciences and Technologies, University of Cagliari, Cagliari, Italy; 6 Epidemiology, Biostatistics and Clinical Trials and Environmental Chemistry Units, Department of Cancer Epidemiology and Prevention, National Cancer Research Institute, Genoa, Italy

**Keywords:** benzene, biomarkers, low exposures, methylation, mitochondrial DNA copy number

## Abstract

Background: Benzene is an established leukemogen at high exposure levels. Although low-level benzene exposure is widespread and may induce oxidative damage, no mechanistic biomarkers are available to detect biological dysfunction at low doses.

Objectives: Our goals were to determine in a large multicenter cross-sectional study whether low-level benzene is associated with increased blood mitochondrial DNA copy number (mtDNAcn, a biological oxidative response to mitochondrial DNA damage and dysfunction) and to explore potential links between mtDNAcn and leukemia-related epigenetic markers.

Methods: We measured blood relative mtDNAcn by real-time polymerase chain reaction in 341 individuals selected from various occupational groups with low-level benzene exposures (> 100 times lower than the Occupational Safety and Health Administration/European Union standards) and 178 referents from three Italian cities (Genoa, Milan, Cagliari).

Results: In each city, benzene-exposed participants showed higher mtDNAcn than referents: mtDNAcn was 0.90 relative units in Genoa bus drivers and 0.75 in referents (*p* = 0.019); 0.90 in Milan gas station attendants, 1.10 in police officers, and 0.75 in referents (*p*-trend = 0.008); 1.63 in Cagliari petrochemical plant workers, 1.25 in referents close to the plant, and 0.90 in referents farther from the plant (*p*-trend = 0.046). Using covariate-adjusted regression models, we estimated that an interquartile range increase in personal airborne benzene was associated with percent increases in mtDNAcn equal to 10.5% in Genoa (*p* = 0.014), 8.2% (*p* = 0.008) in Milan, 7.5% in Cagliari (*p* = 0.22), and 10.3% in all cities combined (*p* < 0.001). Using methylation data available for the Milan participants, we found that mtDNAcn was associated with *LINE-1* hypomethylation (–2.41%; *p* = 0.007) and *p15* hypermethylation (+15.95%, *p* = 0.008).

Conclusions: Blood MtDNAcn was increased in persons exposed to low benzene levels, potentially reflecting mitochondrial DNA damage and dysfunction.

Benzene is a widespread environmental chemical associated with increased risk of hematological malignancies, particularly with acute nonlymphocytic (myeloid) leukemia [Baan et al. 2009; International Agency for Research on Cancer (IARC) 1982, 1987]. Benzene ranks among the top 20 chemicals for production volume in the United States [Centers for Disease Control and Prevention (CDC) 2006]. Outdoor air contains low levels of benzene from several sources, including gas stations, motor vehicle exhaust, and industrial emissions [Agency for Toxic Substances and Disease Registry (ATSDR) 2011]. Most of the current epidemiologic evidence for benzene-related leukemia risk stems from studies among workers exposed to very high levels of benzene (Smith 2010). Multiple investigations have suggested potential hematotoxicity at levels below the occupational exposure limit of 1 ppm (equivalent to 1,000 ppb or 3,250 µg/m^3^, 8-hr time-weighted average) recommended by the U.S. Occupational Safety and Health Administration (OSHA) and European Union (EU 1997; Forastiere et al. 1994; Lan et al. 2004; OSHA 2003). Nonetheless, uncertainties remain about the effects of benzene at low levels. In particular, as remarked in a recent review of benzene health effects (Smith 2010), epidemiology and animal studies have not yet provided conclusive insights about the shape of the exposure–response relationship, particularly at low doses ≤ 1 ppm in air. In this context, the development and use of mechanism-based biological markers has been suggested to hold substantial value in the risk-assessment process (Albertini et al. 2003; Smith 2010).

An important limitation in the current understanding of benzene carcinogenesis is that mechanisms activated at low doses are still largely undefined (Atkinson 2009). *In vitr*o models have shown that some of the reactive metabolites of benzene, such as phenol, catechol, and hydroquinone, can bind to and damage macromolecules, including DNA (Ross 2000). These reactive metabolites may also generate reactive oxygen species (ROS) that can exacerbate DNA damage (Palackal et al. 2002). Recently, Bollati et al. (2007) showed that low-dose exposure to airborne benzene is associated with alterations in DNA methylation in blood DNA of healthy individuals and that the alterations resemble those found in hematological malignancies including hypomethylation of *LINE-1* and *Alu* repetitive elements, hypermethylation of the *p15* tumor suppressor gene, and hypomethylation of melanoma-associated antigen 1 gene (*MAGEA1*). Global DNA hypomethylation has been consistently demonstrated in recent *in vitro* experiments on hydroquinone-treated human lymphoblastoid cells (Ji et al. 2010). These effects of benzene on DNA methylation have been suggested to result from ROS-induced DNA damage (Baccarelli and Bollati 2009).

Mitochondria are both the major intracellular source and primary target of ROS, which are generated under normal conditions as by-products of aerobic metabolism in animal and human cells (Han et al. 2001). Each human and animal cell contains between several hundred and > 1,000 mitochondria, each carrying 2–10 copies of mitochondrial DNA (mtDNA) (Cavelier et al. 2000). MtDNA copy number (mtDNAcn) is positively correlated with the number and size of mitochondria (Lee and Wei 2000). Compared with nuclear DNA, mtDNA has diminished protective histones and DNA repair capacity and is therefore particularly susceptible to ROS-induced damage. Cells challenged with ROS have been shown to synthesize more copies of their mtDNA and to increase their mitochondrial abundance to compensate for damage and meet the increased respiratory demand required for ROS clearance (Lee and Wei 2000). Conversely, ROS are also generated from the increased mitochondria and can, in turn, cause additional oxidative damage to mitochondria and other intracellular constituents, including DNA, RNA, proteins, and lipids.

In a recent study of 40 Chinese shoe and clothing manufacturing workers (Shen et al. 2008), individuals exposed to benzene levels > 1 ppm exhibited higher mtDNAcn in peripheral blood leukocytes than subjects with lower exposure. MtDNAcn has never been studied in larger studies, particularly at the levels of exposure often found in populations in North America and Europe. In the present work, we conducted a multicenter cross-sectional study in Italian cities on individuals exposed to low-level benzene from a variety of sources to examine whether low doses of benzene exposure cause alterations in mtDNAcn.

## Methods

*Study population.* We enrolled 519 participants from three Italian cities (Genoa, Milan, Cagliari). In each city, we included individuals with low-level benzene exposures along with referents. Exposed subjects were selected from occupational categories that entail exposure to low levels of benzene (Fustinoni et al. 2005; Merlo et al. 2003), including 153 bus drivers in Genoa; 78 gas station attendants and 77 police officers in Milan; and 33 workers in a modern petrochemical plant in Cagliari. In Genoa and Milan, referents were occupationally active people from the same area as the exposed participants. In Cagliari, we selected referents who were residents of two small towns located at 2 and 5 km from the petrochemical plant (close referents). An additional sample of referents (distant referents) was selected from an area farther (≥ 20 km) from the plant. Both exposed and referent individuals had been actively employed for ≥ 1 year. We used the same standardized procedures for recruiting all exposed and unexposed individuals in all cities. The smaller numbers of referents compared with exposed workers was determined based on the balance between the efforts required to motivate and recruit referents versus loss in statistical power. A standardized, structured, self-administered questionnaire was used to collect information on lifestyle and risk factors. All participants provided written informed consent to the study, which was approved by the local institutional review boards.

*Personal exposure assessment.* Personal exposure to airborne benzene was determined using passive samplers worn by the study participants near the breathing zone during their work shifts for 5–6 hr (approximately 0800–0930 hours to 1300–1430 hours). In Milan and Genoa, we used passive samplers (stainless-steel tube, 9-mm internal diameter, 90-mm length) containing Chromosorb 106® (Celite Corp., Lompoc, CA, USA) and equipped with a diffusion chamber (Brown 1999). At the end of the monitoring period, the passive sampler was closed with a brass cap and nut, equipped with a polyperfluoroethylene ferule, and kept at –20°C until analysis, performed by thermal desorption followed by gas chromatography/flame ionization detector analysis (Fustinoni et al. 2005). In Cagliari, we used Radiello® passive samplers, equipped with a 35- to 50-mesh charcoal cartridge (Supelco, Sigma-Aldrich, Milan, Italy). At the end of sampling, the cartridge was sealed in glass tubes and kept in a clean box at room temperature until gas chromatography/mass spectrometry analysis (Fustinoni et al. 2010a), which occurred within 30 days from collection, as per manufacturer’s instructions. The two sampling methods have been shown previously to have similar recovery performances (Hayes 2009). All benzene analyses were performed at the Environmental Chemistry Unit of the National Cancer Research Institute (Genoa, Italy). The detection limit for airborne benzene was 6 µg/m^3^ (1.85 ppb). Sixty-five individuals (12.5%) had benzene levels below the detection limit (DL) and were assigned a value corresponding to DL/_√_^–^2 (Hornung and Reed 1990).

*mtDNAcn analysis.* Total DNA was extracted using the Wizard Genomic DNA purification kit (Promega Corporation, Madison, WI, USA) from whole blood collected in EDTA tubes at the beginning of the work shift. Relative mtDNAcn was measured by quantitative real-time polymerase chain reaction (PCR), as described previously (Hou et al. 2010). All samples were run in triplicates in 384-well plates on a 7900HT Fast Real-Time PCR System (Applied Biosystems, Foster City, CA, USA). The assay is based on the ratio of copy number estimates of a mitochondrial gene (*mtND1*) to those of a nuclear gene [human beta globin (*hbg*)]. The *mtND1*/*hbg* ratio thus calculated in experimental samples is then scaled to a standard DNA sample to obtain relative mtDNAcn values controlled for plate effects. The standard DNA sample was obtained by pooling DNA from 20 participants randomly selected from the Milan referents and was used to generate a fresh five-point standard curve (range: 20–0.247 ng) in every *mtND1* and *hbg* run. Primers and conditions for mtDNAcn analysis are provided in the Supplemental Material (http://dx.doi.org/10.1289/ehp.1103979).

In addition to the mtDNAcn data, DNA methylation measures on blood DNA by PCR-pyrosequencing were available for the subset of Milan participants as part of previous work evaluating the effects of benzene exposure on DNA methylation (Bollati et al. 2007).

*Statistical analysis.* We used standard descriptive statistics [means, SDs, medians, interquartile ranges (IQRs), and proportions] to summarize data. MtDNAcn showed asymmetric distributions within each city and exposure group and was log-transformed to approximate normality. Throughout this paper, we report geometric means (GMs) and corresponding 95% confidence intervals (CIs). Differences in mtDNAcn across exposure groups were evaluated using one-way ANOVA and tests for trend computed via linear regression analysis. In addition, we evaluated the association between mtDNAcn and exposure groups by fitting multivariate models adjusted for age (continuous), sex (male, female), smoking (never, former, current smoker), and number of cigarettes/day (continuous). We used linear regression models to examine the association of airborne benzene levels with mtDNAcn. Scatterplots of airborne benzene versus mtDNAcn showed a nonlinear relationship that approximated linearity when both variables were log-transformed. All models were thus fitted by regressing log[mtDNAcn] over log[benzene]. To exclude confounding by factors associated with differences across cities (including potential differences from the different protocol used for air benzene sampling in Cagliari), we first fitted unadjusted and adjusted models for each of the cities separately, and then we fitted models for all participants combined. In the models of all participants combined, we fitted an independent indicator variable for each of the cities (Genoa, Milan, Cagliari) in both unadjusted and adjusted models. In both city-specific and combined analyses, adjusted models included age, sex, smoking, and number of cigarettes/day as independent variables. To facilitate understanding of effect sizes, effects are expressed throughout this paper as percent variation in mtDNAcn per IQR increase in benzene exposure. To confirm the results from multiple linear regression models evaluating the association between relative mtDNAcn and airborne benzene, we performed a set of sensitivity analyses [for details, see Supplemental Material (http://dx.doi.org/10.1289/ehp.1103979)].

In the subset of the study participants with DNA methylation data (*n* = 212), we fitted multiple regression models (adjusted for age, sex, smoking, and number of cigarettes/day) to evaluate the association of log[mtDNAcn] with *LINE-1*, *Alu*, *p15*, or *MAGEA1* DNA methylation. DNA methylation variables were also log-transformed to approximate normality; effects are expressed as percent variation in DNA methylation per IQR change in mtDNAcn.

Outliers were excluded from all regression analyses by dropping observations with studentized residuals that exceeded ± 3. Using these criteria, a variable number of observations (between one and seven) was dropped from each model. All tests of statistical significance were two-sided.

Statistical analyses were performed using Stata/MP 11.1 (StataCorp, College Station, TX, USA), R (R Foundation for Statistical Computing, Vienna, Austria), and SAS 9.2 (SAS Institute Inc., Cary, NC, USA).

## Results

*Study population.* Table 1 summarizes the characteristics of the study population by city and exposure group. The overall median age was 39 years of age considering all participants, with median values ranging from a minimum of 30 years of age (Milan police officers) to a maximum of 55 years of age (Cagliari distant referents). In all cities combined, males represented 81% of the study population. The proportion of overall current smokers (32%) was similar to the proportion of smokers in the Italian male adult population [Istituto nazionale di statistica (ISTAT) 2009]. The mean number of cigarettes/day was 16 for all participants combined. Most of the study participants (66%) resided in the suburbs at the time of enrollment.

**Table 1:** Characteristics of the study population.

Sex			Smoking					Cigarettes per day*c*	Home address*a*		
City	Exposure group	*n*	Age*b*	Male	Female	Never	Former		Current	City	Suburbs
Genoa		Referents		49		42 (9)		47 (96)		2 (4)		25 (51)		11 (22)		13 (27)		16 ± 7		23 (47)		26 (53)
		Bus drivers		153		38 (23)		150 (98)		3 (2)		61 (40)		45 (29)		47 (31)		17 ± 9		50 (33)		103 (67)
Milan		Referents		57		36 (18)		38 (67)		19 (33)		26 (46)		8 (14)		23 (40)		15 ± 10		19 (33)		38 (67)
		Police officers		77		30 (8)		47 (61)		30 (39)		40 (52)		9 (12)		28 (36)		15 ± 7		10 (13)		65 (87)
		Gas station attendants		78		41 (18)		69 (88)		9 (12)		30 (38)		16 (21)		32 (41)		17 ± 8		27 (35)		51 (65)
Cagliari		Distant referents		16		55 (15)		10 (62)		6 (38)		8 (50)		6 (38)		2 (12)		13 ± 4		9 (56)		7 (44)
		Close referents		56		46 (17)		27 (48)		29 (52)		31 (55)		15 (27)		10 (18)		16 ± 6		21 (38)		35 (62)
		Petrochemical workers		33		36 (21)		32 (97)		1 (3)		10 (30)		10 (30)		13 (40)		15 ± 9		16 (48)		17 (52)
All subjects				519		39 (16)		420 (81)		99 (19)		231 (45)		120 (23)		168 (32)		16 ± 8		175 (34)		342 (66)
Values are *n* (%) unless noted otherwise. **a**The counts do not add up to the total number of participants because of two missing values. **b**Median (IQR). **c**Mean ± SD; number of cigarettes/day calculated among current smokers only.																						

*Exposure levels to airborne benzene.* Across all cities, airborne benzene exposure levels as measured on personal samplers were higher for individuals in the exposed groups than the referents (Table 2). Among the occupationally exposed participants, the highest exposure levels were observed in the Milan gas station attendants [GM = 69.9 µg/m^3^; 95% CI: 57.4, 85.2 (21.5 ppb; 95% CI: 17.7, 26.2)], followed by the Cagliari petrochemical workers [GM = 35.4 µg/m^3^; 95% CI: 20.5, 61.0 (10.9 ppb; 95% CI: 6.31, 18.8)]. GMs across different referent groups were all between 5.9 µg/m^3^ (1.82 ppb, Cagliari distant referents) and 8.7 µg/m^3^ (2.68 ppb, Genoa referents).

**Table 2:** Airborne benzene by city and exposure group.

Benzene exposure levels (µg/m^3^)*a*													
City	Exposure group	Minimum	25th percentile	Median	75th percentile	Maximum	GM (95% CI)		*p*-Value
Genoa		Referents		4.2		4.2		8.6		13.8		45.8		8.7	(7.3, 10.5)		
		Bus drivers		4.2		14.8		20.5		30.9		92.1		20.5	(18.7, 22.4)		< 0.001*b*
Milan		Referents		4.2		4.2		6.3		12.8		57.1		8.1	(6.6, 10.0)		
		Police officers		9.03		19.0		21.8		31.1		315.7		25.0	(22.0, 28.3)		
		Gas station attendants		11.5		37.9		60.9		130.9		477.9		69.9	(57.4, 85.2)		< 0.001*c*
Cagliari		Distant referents		4.2		4.2		6.0		7.0		9.0		5.9	(4.9, 7.1)		
		Close referents		4.2		5.1		8.0		11.0		27.0		8.2	(7.1, 9.4)		
		Petrochemical workers		6.0		11.0		25.0		63.0		1250.0		35.4	(20.5, 61.0)		< 0.001*c*
**a**To convert micrograms per cubic meter to parts per billion, divide by 3.25. **b**Mann-Whitney U nonparametric test for difference between referents and bus drivers. **c**Nonparametric test (Cuzick) for trend across exposure categories.																	

*Relative mtDNAcn by exposure groups.* In each city, exposed participants had consistently higher mtDNAcn levels than referents (Table 3). In Genoa, mean relative mtDNAcn was 0.75 (95% CI: 0.66, 0.85) in referents and 0.90 (95% CI: 0.84, 0.97) in bus drivers (*p* = 0.019) in analysis adjusted for age, sex, smoking, and number of cigarettes/day. In Milan, adjusted mean relative mtDNAcn was 0.75 (95% CI: 0.69, 0.82) in referents, 1.10 (95% CI: 1.01, 1.19) in police officers, and 0.90 (95% CI: 0.83, 0.98) in gas station attendants (*p*-trend = 0.008). In Cagliari, adjusted mean relative mtDNAcn was 0.90 (95% CI: 0.60, 1.41) in distant referents, 1.25 (95% CI: 1.03, 1.51) in close referents, and 1.63 (95% CI: 1.22, 2.18) in petrochemical workers (*p*-trend = 0.046). Referents exhibited different mtDNAcn across cities, with higher mean levels in Cagliari compared with Milan and Genoa (Table 3).

**Table 3:** Relative mtDNAcn by city and exposure group.

MtDNAcn (unadjusted)						MtDNAcn (adjusted)					
City	Exposure group	*n*	Mean**(95% CI)*a*		*p-*Value*b*	*p*-Trend*c*	Mean**(95% CI)*d*		*p-*Value*b*	*p-*Trend*c*
Genoa		Referents		48		0.75	(0.65, 0.86)						0.75	(0.66, 0.85)				
		Bus drivers		151		0.90	(0.84, 0.97)		0.013		—		0.90	(0.84, 0.97)		0.019		—
Milan		Referents		56		0.76	(0.68, 0.84)						0.75	(0.69, 0.82)				
		Police officers		77		1.14	(1.07, 1.22)		< 0.001				1.10	(1.01, 1.19)		< 0.001		
		Gas station attendants		76		0.86	(0.79, 0.94)		0.037		0.180		0.90	(0.83, 0.98)		0.005		0.008
Cagliari		Distant referents		10		0.94	(0.59, 1.48)						0.90	(0.60, 1.41)				
		Close referents		47		1.24	(1.01, 1.52)		0.215				1.25	(1.03, 1.51)		0.206		
		Petrochemical workers		24		1.64	(1.30, 2.07)		0.024		0.020		1.63	(1.22, 2.18)		0.041		0.046
**a**GM and 95% CI. **b**One-way ANOVA for difference versus referents. **c**Linear regression analysis for test for trend across exposure categories. **d**GM and 95% CI adjusted for age, sex, smoking (never, former, current), number of cigarettes/day.																		

**Figure 1:**
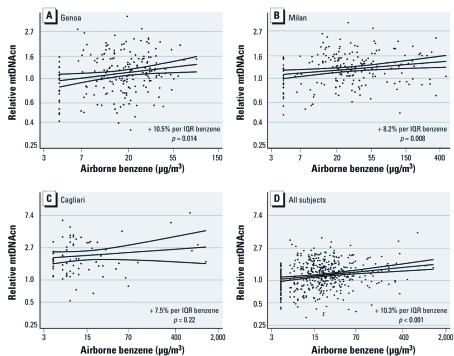
Association between relative mtDNAcn and airborne benzene. Scatterplots of mtDNAcn versus airborne benzene levels for Genoa (*A*), Milan (*B*), and Cagliari (*C*), and for all participants combined (*n* = 519; *D*). Covariate-adjusted percent changes in mtDNAcn estimated per IQR increase in personal airborne benzene are shown.

*Relative mtDNAcn and airborne benzene.* Relative mtDNAcn showed a positive correlation with airborne benzene concentrations in each of the cities (Figure 1A–C). In multivariate regression models adjusted for age, sex, smoking, and number of cigarettes/day, we found a significant 10.5% increase (95% CI: 2.1, 19.6; *p* = 0.014) in relative mtDNAcn per IQR benzene increase in Genoa (Figure 1A) and a significant 8.2% increase (95% CI: 2.2, 14.7; *p* = 0.008) in Milan (Figure 1B). In Cagliari, the correlation between relative mtDNAcn and airborne benzene was also positive (7.5% increase; 95% CI: –4.2, 20.6) (Figure 1C), but not statistically significant (*p* = 0.22). City-specific unadjusted analyses showed similar results, except for Milan, where the percent increase was lower than in the multivariate analyses (10.7% increase; 95% CI: 2.4, 19.7; *p* = 0.011 in Genoa; 3.3% increase; 95% CI: –2.7, 9.7; *p* = 0.29 in Milan; 9.0% increase; 95% CI: –1.6, 20.8; *p* = 0.10 in Cagliari). Analyses on all participants combined (Figure 1D) showed a highly significant increase in relative mtDNAcn associated with benzene levels for both unadjusted (7.8% increase; 95% CI: 2.9, 13.0; *p* = 0.002) and adjusted regressions (10.3% increase; 95% CI: 5.4, 15.5; *p* < 0.001).

**Figure 2:**
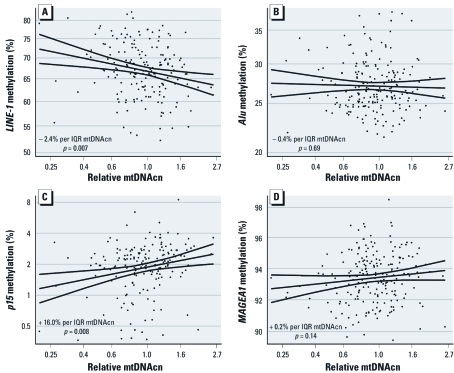
Association between DNA methylation and relative mtDNAcn. Scatterplots of *LINE-1* (*A*), *Alu* (*B*), *p15* (*C*), or *MAGEA1* (*D*) methylation versus mtDNAcn in the Milan subset of the study population (*n* = 212). Covariate-adjusted percent changes in DNA methylation estimated per IQR increase in mtDNAcn are shown.

*DNA methylation and relative mtDNAcn.* Taking advantage of extant epigenetic data in the Milan subset of the study population (*n* = 212), we also explored whether mtDNAcn was associated with DNA methylation in *LINE-1*, *Alu*, *p15*, and *MAGEA1* by multivariate regression models (adjusted for age, sex, smoking, and number of cigarettes/day). Correlations of mtDNAcn with DNA methylation measures are shown in Figure 2A–D. *LINE-1* methylation showed a significant decrease associated with an IQR increase in relative mtDNAcn (–2.4% change; 95% CI: –4.1, –0.7; *p* = 0.007) (Figure 2A); *p15* methylation exhibited a significant percent increase (16.0% change; 95% CI: 4.1, 29.2; *p* = 0.008) (Figure 2C); *Alu* and *MAGEA1* methylation (Figure 2B,D) did not show significant variations correlated with increasing relative mtDNAcn (–0.4% change; 95% CI: –2.5, 1.7; *p* = 0.69 for *Alu*; 0.2% change; 95% CI: –0.1, 0.6; *p* = 0.14 for *MAGEA1*). Unadjusted analyses showed very similar results (–2.3% change; 95% CI: –3.9, –0.7; *p* = 0.005 for *LINE-1*; 13.0% change; 95% CI: 2.3, 24.8; *p* = 0.017 for *p15*; 0.4% change; 95% CI: –1.5, 2.4; *p* = 0.69 for *Alu*; 0.3% change; 95% CI: –0.1, 0.6; *p* = 0.06 for *MAGEA1*).

## Discussion

In this multicity investigation of individuals with low-level exposure to benzene, we found that blood mtDNAcn was increased in association with airborne benzene exposure. We observed differences in mtDNA levels by comparing different exposure groups within each city. We demonstrated a dose–response relationship between mtDNAcn and benzene exposure levels within each city and overall using personal sampler data as direct measures of individual benzene exposure. In the Milan study participants, increasing mtDNAcn correlated with decreased *LINE-1* methylation and increased *p15* methylation.

Benzene exposure has been linked consistently with hematological malignancies (Baan et al. 2009; IARC 1982, 1987) in cohorts of workers occupationally exposed to levels substantially higher than those found in the environment or even in modern facilities where appropriate occupational safety procedures are implemented. In our study population, benzene exposure levels (median = 19.2 µg/m^3^, equivalent to 6 ppb) were more than 100 times lower, on average, than the occupational standard limit of 1 ppm (equivalent to 1,000 ppb or 3,250 µg/m^3^) set by OSHA/EU (EU 1997; OSHA 2003). Even in the most-exposed participant, benzene exposure level (1,250 µg/m^3^, 380 ppb) was still about one third of the occupational exposure limit.

MtDNAcn has been associated with benzene exposure in Chinese workers exposed to levels of benzene (14.3 ± 20.4 ppm, reported mean ± SD) comparable with those associated with hematological malignancies (Shen et al. 2008). Our study indicates that mtDNAcn alterations can be observed also at low exposure doses, possibly reflecting the activation of key cellular processes such as oxidative stress, which is known to operate in early carcinogenesis. Increased mtDNAcn has been suggested to have a dual role in cells challenged by oxidative stress. On one hand, it stimulates mitochondrial proliferation to supply energy to meet the need for cell survival, including damage repair and synthesizing new proteins (Lee and Wei 2000). On the other hand, the increasing abundance of dysfunctional mitochondria causes excess ROS production and further oxidative damage that may initiate cell senescence or death (Lee and Wei 2005). Baccarelli and Bollati (2009) demonstrated that alterations in DNA methylation can also result from oxidative insults. Aberrant DNA methylation, including hypomethylation of repetitive elements and hypermethylation of tumor suppressor genes, is increasingly recognized as a critical step in malignant transformation (Rodriguez-Paredes and Esteller 2011). In particular, *LINE-1* hypomethylation and *p15* hypermethylation are commonly found in acute nonlymphocytic leukemia and other hematological malignancies (Deneberg et al. 2010). Using methylation data available to us from a previous investigation (Bollati et al. 2007) of the Milan study participants, we found a linear association of mtDNAcn with both *LINE-1* hypomethylation and *p15* hypermethylation, whereas no association was observed between mtDNAcn and *Alu* or *MAGEA1* methylation. Hypomethylation of *LINE-1*, which has often been used as a surrogate for global methylation, is believed to contribute to determining chromosomal instability and breakage. The tumor suppressor gene *p15* shows low or no methylation in normal cells, whereas it is hypermethylated in acute nonlymphocytic leukemia cells (Claus and Lubbert 2003) as well as in other hematological malignancies (Galm et al. 2006). *p15* encodes a cyclin-dependent kinase inhibitor, which functions as a cell growth regulator controlling cell cycle G1 progression (Hannon and Beach 1994). Additionally, *p15* hypermethylation is widely considered to contribute to the loss of cell cycle arrest responses in malignant cells (Herman et al. 1996; Ng et al. 1997). Our findings on *LINE-1* methylation follow the same direction as a recent investigation of the Normative Aging Study cohort (Baccarelli et al. 2009) that showed an association between exposure to air pollution from traffic particles and DNA methylation of *LINE-1*, but no association with *Alu* methylation. Even though *LINE-1* and *Alu* repetitive element methylation has been demonstrated to correlate with global DNA methylation in cancer tissues (Weisenberger et al. 2005), the two repetitive elements are controlled through different mechanisms and might respond differently to oxidative stress (Bollati et al. 2009). We did not observe any association between mtDNAcn and *MAGEA1* methylation. We surmise that *MAGEA1* may be part of a benzene-induced pathway that does not directly involve oxidative stress. Moreover, in our previous work on effects of benzene exposure on DNA methylation (Bollati et al. 2007), we found only a weak, borderline significant association between benzene exposure and *MAGEA1* methylation.

Whether mtDNAcn has a direct role in carcinogenesis is still under investigation. Recent longitudinal studies have shown that individuals with higher blood mtDNAcn at baseline have higher risk of developing non-Hodgkin lymphoma and lung cancer (Hosgood et al. 2010; Lan et al. 2008). In addition, mtDNAcn alterations are associated with impaired apoptosis and subsequent increased cellular proliferation (Eliseev et al. 2003) as well as with nuclear DNA mutations after mtDNA insertion into the genome (Hazkani-Covo et al. 2010). Although these results are suggestive of potential roles of mtDNAcn in carcinogenesis, whether mtDNAcn alterations contribute to determining increased risks of malignancies in benzene-exposed individuals remains to be determined.

We note that at least some of our findings might be explained by exposure to co-pollutants whose levels may track together with airborne benzene levels. In all the cities in the present study, exposed individuals had higher mtDNAcn levels than referents. However, police officers in Milan who had exposure levels that were intermediate between gas station attendants and referents showed higher mtDNAcn levels than the gas station attendants. Conversely, Milan gas station attendants, who were the exposure group with the highest exposure across cities, were the group with the lowest mtDNAcn among exposed subjects. Although police officers and gas station attendants are both exposed to environmental benzene, these occupational categories work in different exposure settings. Gas station attendants are exposed mainly to benzene vapors during filling operations, whereas police officers receive most of their exposure from vehicular combustion by-products (Cattaneo et al. 2010). Combustion by-products from traffic include not only benzene but also particulate matter and nitric oxide, all of which can contribute to generating oxidative stress (Risom et al. 2005). Because particulate matter and nitric oxide exposures were not measured in our study, we cannot determine their possible contributions to the increased mtDNAcn among the Milan police officers. Levels of mtDNAcn in the referent group in Cagliari were higher than those found in the Milan and Genoa referents. Cagliari is located on Sardinia, whose inhabitants mostly have a unique genetic background that goes back approximately 8,000 years to the island’s original settlers (NIA 2009; Pilia et al. 2006). Consequently, Sardinians have genetic characteristics that are remarkably different from individuals living in other Italian regions (Calò et al. 2008). Using a twin study design, Xing et al. (2008) demonstrated that mtDNA content is a trait with high genetic heritability. Based on these observations, the higher mtDNAcn values in the Cagliari participants might be determined by the different genetic background of the study individuals.

We used the same standardized study procedures across the three cities, including uniform questionnaires, data and blood collection, and mtDNAcn analysis. Air benzene sampling was performed using Chromosorb 106® stainless steel passive samplers (Celite Corp.) in Milan and Genoa, and Radiello® passive samplers (Supelco) in Cagliari. A head-to-head comparison of the two different passive samplers showed no difference in recovery performances (Hayes 2009). The populations of the three cities in our study likely differ for multiple factors, including but not limited to lifestyle, diet, and climatic conditions. To take into account these differences and avoid potential confounding, we first analyzed each city separately. Analyses on all participants combined were then conducted by fitting regression models that included an independent indicator variable for each of the cities.

Our study has the advantage of relying on personal measurements of benzene exposure obtained from portable passive samplers. Although limited to 1 day of airborne benzene sampling, airborne benzene measures showed significant differences between the exposure groups and were therefore used as a proxy for the usual exposure of the study participants in this cross-sectional study. In a new recruitment campaign, we recently reevaluated a subset of 53 Milan study participants (18 referents and 35 gas station attendants) from the original study and found high correlations of benzene concentration between the original study and current follow-up (Fustinoni et al. 2010b). Exposure levels in the exposed groups were comparable with those reported in previous studies on individuals in similar occupations (Angelini et al. 2011; Cattaneo et al. 2010; Lovreglio et al. 2010; Ruchirawat et al. 2010). We used a relative measure of mtDNAcn, which has been shown to be highly precise and reproducible (Xing et al. 2008). This method, which has been widely used in large human studies (Shen et al. 2010; Xing et al. 2008), expresses mtDNAcn as a relative measure in relation to standard DNA. Although this method is well suited for comparisons between groups that are usually all scaled to the same standard DNA, as in our study, the use of different standard DNA samples in different studies may limit external comparability.

## Conclusions

Our investigation of individuals with low-level exposure to benzene in three Italian cities showed increased mtDNAcn in association with airborne benzene exposure. Whether mitochondrial damage and dysfunction potentially related with increased mtDNAcn reflects the risk of hematological malignancies due to low-dose benzene remains to be determined in future prospective investigations.

## Supplemental Material

(164 KB) PDF
