# Supplementary material for: Increased Mitochondrial DNA Copy Number in Occupations Associated with Low-Dose Benzene Exposure
Source: Environ Health Perspect. 2011 Oct 17;120(2):210–5. doi: 10.1289/ehp.1103979 (PMC3279451; doi:10.1289/ehp.1103979)
Supplement: (164 KB) PDF [file ehp.1103979.s001.pdf]

SUPPLEMENTAL MATERIAL

Increased Mitochondrial DNA Copy Number in Occupations Associated with Low-Dose Benzene Exposure

Michele Carugno[1], Angela Cecilia Pesatori[1, 2], Laura Dioni[1], Mirjam Hoxha[1], Valentina Bollati[1], Benedetta Albetti[1], Hyang-Min Byun[3], Matteo Bonzini[4], Silvia Fustinoni[2], Pierluigi Cocco[5], Giannina Satta[5], Mariagrazia Zucca[5], Domenico Franco Merlo[6], Massimo Cipolla[6], Pier Alberto Bertazzi[1, 2], Andrea Baccarelli[3]

[1]Department of Occupational and Environmental Health, Università degli Studi di Milano, Milan, Italy;

[2]Fondazione IRCCS Ca' Granda - Ospedale Maggiore Policlinico, Milan, Italy;

[3]Department of Environmental Health, Harvard School of Public Health, Boston, Massachusetts, USA;

[4]Department of Clinical and Biological Sciences, University of Insubria, Varese, Italy;

[5]Department of Biomedical Sciences and Technologies, University of Cagliari, Cagliari, Italy

[6]Epidemiology, Biostatistics and Clinical Trials & Environmental Chemistry Units, Department of Cancer Epidemiology and Prevention, National Cancer Research Institute, Genoa, Italy.

Table of Contents

Primers and Conditions for Mitochondrial DNA Copy Number (mtDNAcn) Analysis p S2

Sensitivity Analyses p S3

References Quoted in the Supplemental Material p S4

Primers and Conditions for Mitochondrial DNA Copy Number (mtDNAcn) analysis

The primers for RT Q-PCR analysis of mtND1 were: mtND1-F 5'CAC CCA AGA ACA GGG TTT GT3', and mtND1-R 5'TGG CCA TGG GTA TGT TGT TA3' (Lin et al. 2008). The primers for RT Q-PCR analysis of hbg were: hbg-F 5'GCT TCT GAC ACA ACT GTG TTC ACT AGC3', and hbg-R 5'CAC CAA CTT CAT CCA CGT TCA CC3'. The mtND1 PCR mixture in a total of 10 µl contained: iQ SYBR Green Supermix 1x (Bio-Rad Laboratories, Hercules, California, USA), MtF3212 500 nM, MtR3319 500 nM, EDTA 1x. The S (hbg) PCR mixture in a total of 10 µl contained: iQ SYBR Green Supermix 1x (Bio-Rad Laboratories, Hercules, California, USA), hbg-Forward 500 nM, hbg-Reverse 500 nM, EDTA 1x. We loaded 4 ng DNA in each PCR reaction. The thermal cycling conditions for mtND1 PCR were: initial 2 minutes at 50 °C, and 3 minutes at 95 °C to activate the hot-start iTaq DNA polymerase, followed by 35 cycles comprised of 15 seconds denaturation at 95 °C and 60 seconds anneal/extend at 60 °C. The thermal cycling conditions for the hbg PCR were: 3 minutes at 95 °C to activate the hot-start iTaq DNA polymerase, followed by 35 cycles comprised of 15 seconds denaturation at 95 °C and 60 seconds anneal/extend at 58 °C. The coefficient of variation for the Mt/S ratio in duplicate samples analyzed on two different days was 7.8%.

Sensitivity Analyses

Sixty-five of the 519 (12.5%) study participants had airborne benzene below the analytical limit of detection of the method (6 µg/m^3, 1.85 ppb) and were assigned in our primary analysis a value corresponding to the detection limit divided by the square root of two. As a sensitivity analysis, we fitted multiple linear regression models using multiple imputations to replace each airborne benzene value below 6 µg/m^3 (non-detects) with several values that represent the uncertainty about which value to impute (Rubin 1976, 1987). Multiple imputations were performed with the "Markov Chain Monte Carlo" method using the PROC MI and PROC MIANALYZE SAS procedure. In this procedure multiple datasets are created by repeating the imputation for non-detects and, from each of them, parameter estimates and covariances are obtained using standard analyses. These estimates are then combined and the total variance of the final estimate is computed. The analysis based on multiple imputations confirmed the correlation between airborne benzene and mtDNAcn observed in our primary analysis. For instance, the percent variations in mtDNAcn for the adjusted model on all participants combined were very similar between multiple imputation (10.1% increase, 95%CI 5.0-15.3, p<0.001 for an IQR exposure increase) and the primary analysis (10.3% increase, 95%CI 5.4-15.5, p<0.001 for an IQR exposure increase).

We also explored an alternative method to account for city effects in the combined analyses by using mixed effect models fitting a random intercept for each city instead of the main effect indicator variable used in the primary analysis. The results of the mixed effect models were very similar to those from linear regression models (10.2% increase in mtDNAcn, 95%CI 5.3-15.4, p<0.001 for an IQR exposure increase).

To explore departure from linearity of the association between relative mtDNAcn and airborne benzene, we fitted a penalized regression spline for the exposure variable (log[benzene]) and used generalized cross-validation (GCV) to select the spline parameters

for its relation with the dependent variable (log[mtDNAcn]). This model did not show major departures from the linear model: the GCV algorithm selected approximately one degree of freedom for the smoothed parameter relative to log[benzene].

References Quoted in the Supplemental Material

Lin CS, Wang LS, Tsai CM, Wei YH. 2008. Low copy number and low oxidative damage of mitochondrial DNA are associated with tumor progression in lung cancer tissues after neoadjuvant chemotherapy. Interact Cardiovasc Thorac Surg 7(6):954-958.

Rubin DB. 1976. Inference and missing data. Biometrika 63:581–592.

Rubin DB. 1987. Multiple Imputation for Nonresponse in Surveys. John Wiley, New York. John Wiley, New York.
